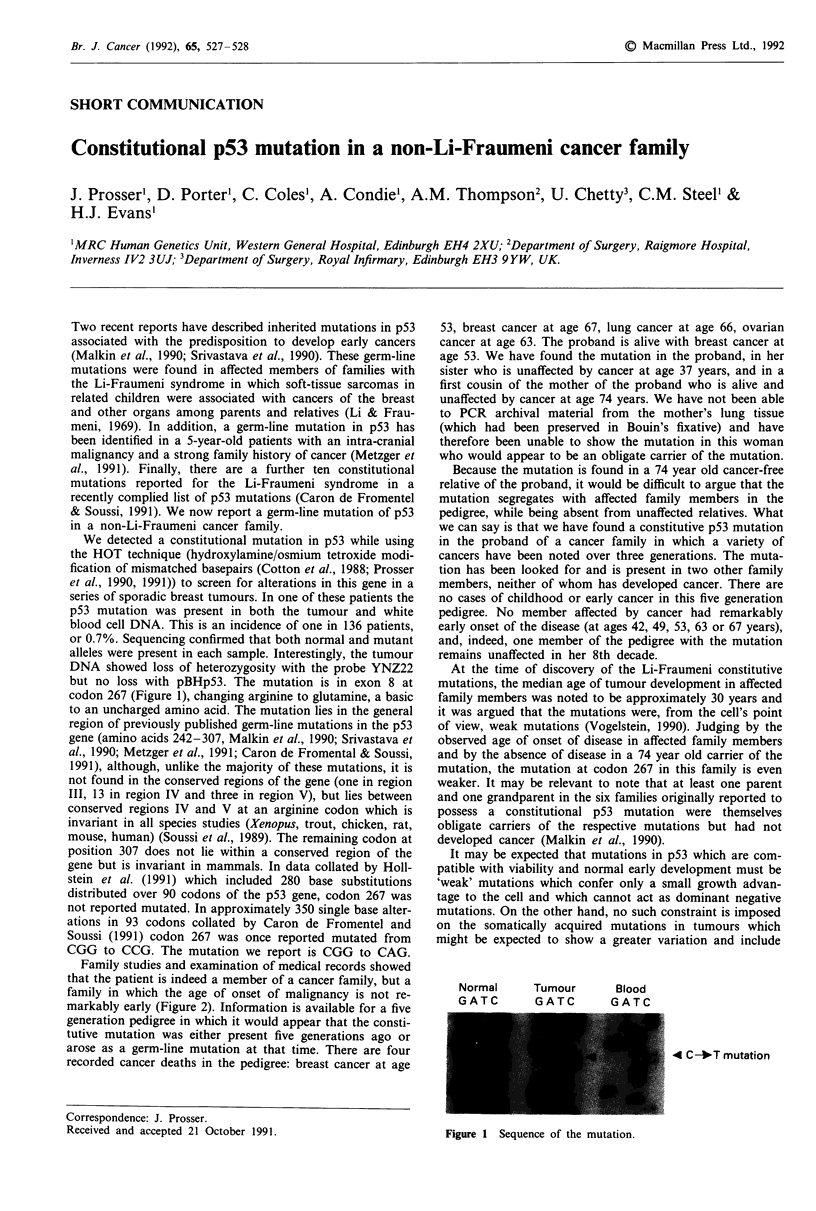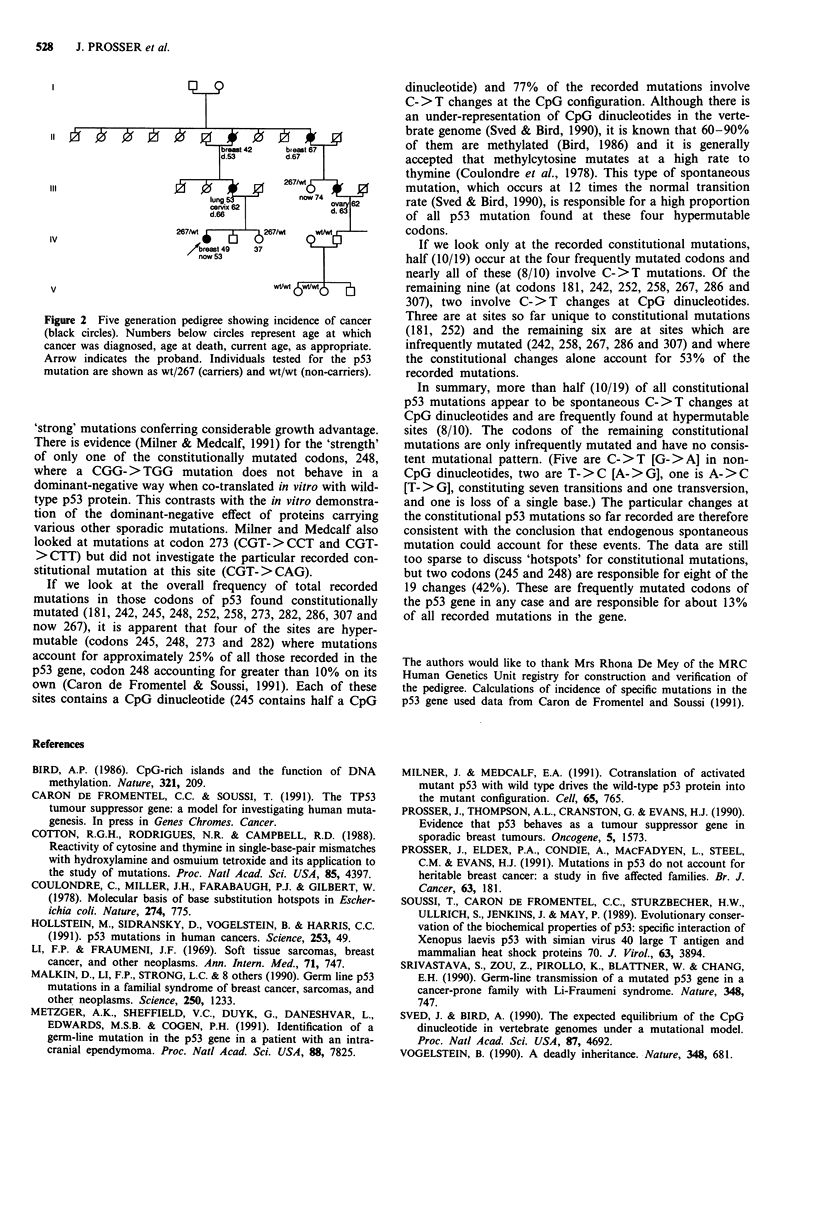# Constitutional p53 mutation in a non-Li-Fraumeni cancer family.

**DOI:** 10.1038/bjc.1992.109

**Published:** 1992-04

**Authors:** J. Prosser, D. Porter, C. Coles, A. Condie, A. M. Thompson, U. Chetty, C. M. Steel, H. J. Evans

**Affiliations:** MRC Human Genetics Unit, Western General Hospital, Edinburgh, UK.

## Abstract

**Images:**


Br. J. Cancer (1992), 65, 527-528 © Macmillan Press Ltd., 1992

SHORT COMMUNICATION

### Constitutional p53 mutation in a non-Li-Fraumeni cancer family

J. Prosser[1], D. Porter[1], C. Coles[1], A. Condie[1], A.M. Thompson[2], U. Chetty[3], C.M. Steel[1] & H.J. Evans[1]

[1]*MRC Human Genetics Unit, Western General Hospital, Edinburgh EH4 2XU;* [2]*Department of Surgery, Raigmore Hospital, Inverness IV2 3UJ;* [3]*Department of Surgery, Royal Infirmary, Edinburgh EH3 9YW, UK.*

Two recent reports have described inherited mutations in p53 associated with the predisposition to develop early cancers (Malkin *et al.*, 1990; Srivastava *et al.*, 1990). These germ-line mutations were found in affected members of families with the Li-Fraumeni syndrome in which soft-tissue sarcomas in related children were associated with cancers of the breast and other organs among parents and relatives (Li & Fraumeni, 1969). In addition, a germ-line mutation in p53 has been identified in a 5-year-old patients with an intra-cranial malignancy and a strong family history of cancer (Metzger *et al.*, 1991). Finally, there are a further ten constitutional mutations reported for the Li-Fraumeni syndrome in a recently complied list of p53 mutations (Caron de Fromentel & Soussi, 1991). We now report a germ-line mutation of p53 in a non-Li-Fraumeni cancer family.

We detected a constitutional mutation in p53 while using the HOT technique (hydroxylamine/osmium tetroxide modification of mismatched basepairs (Cotton *et al.*, 1988; Prosser *et al.*, 1990, 1991)) to screen for alterations in this gene in a series of sporadic breast tumours. In one of these patients the p53 mutation was present in both the tumour and white blood cell DNA. This is an incidence of one in 136 patients, or 0.7%. Sequencing confirmed that both normal and mutant alleles were present in each sample. Interestingly, the tumour DNA showed loss of heterozygosity with the probe YNZ22 but no loss with pBHp53. The mutation is in exon 8 at codon 267 (Figure 1), changing arginine to glutamine, a basic to an uncharged amino acid. The mutation lies in the general region of previously published germ-line mutations in the p53 gene (amino acids 242-307, Malkin *et al.*, 1990; Srivastava *et al.*, 1990; Metzger *et al.*, 1991; Caron de Fromental & Soussi, 1991), although, unlike the majority of these mutations, it is not found in the conserved regions of the gene (one in region III, 13 in region IV and three in region V), but lies between conserved regions IV and V at an arginine codon which is invariant in all species studies (*Xenopus*, trout, chicken, rat, mouse, human) (Soussi *et al.*, 1989). The remaining codon at position 307 does not lie within a conserved region of the gene but is invariant in mammals. In data collated by Hollstein *et al.* (1991) which included 280 base substitutions distributed over 90 codons of the p53 gene, codon 267 was not reported mutated. In approximately 350 single base alterations in 93 codons collated by Caron de Fromentel and Soussi (1991) codon 267 was once reported mutated from CGG to CCG. The mutation we report is CGG to CAG.

Family studies and examination of medical records showed that the patient is indeed a member of a cancer family, but a family in which the age of onset of malignancy is not remarkably early (Figure 2). Information is available for a five generation pedigree in which it would appear that the constitutive mutation was either present five generations ago or arose as a germ-line mutation at that time. There are four recorded cancer deaths in the pedigree: breast cancer at age 53, breast cancer at age 67, lung cancer at age 66, ovarian cancer at age 63. The proband is alive with breast cancer at age 53. We have found the mutation in the proband, in her sister who is unaffected by cancer at age 37 years, and in a first cousin of the mother of the proband who is alive and unaffected by cancer at age 74 years. We have not been able to PCR archival material from the mother's lung tissue (which had been preserved in Bouin's fixative) and have therefore been unable to show the mutation in this woman who would appear to be an obligate carrier of the mutation.

Because the mutation is found in a 74 year old cancer-free relative of the proband, it would be difficult to argue that the mutation segregates with affected family members in the pedigree, while being absent from unaffected relatives. What we can say is that we have found a constitutive p53 mutation in the proband of a cancer family in which a variety of cancers have been noted over three generations. The mutation has been looked for and is present in two other family members, neither of whom has developed cancer. There are no cases of childhood or early cancer in this five generation pedigree. No member affected by cancer had remarkably early onset of the disease (at ages 42, 49, 53, 63 or 67 years), and, indeed, one member of the pedigree with the mutation remains unaffected in her 8th decade.

At the time of discovery of the Li-Fraumeni constitutive mutations, the median age of tumour development in affected family members was noted to be approximately 30 years and it was argued that the mutations were, from the cell's point of view, weak mutations (Vogelstein, 1990). Judging by the observed age of onset of disease in affected family members and by the absence of disease in a 74 year old carrier of the mutation, the mutation at codon 267 in this family is even weaker. It may be relevant to note that at least one parent and one grandparent in the six families originally reported to possess a constitutional p53 mutation were themselves obligate carriers of the respective mutations but had not developed cancer (Malkin *et al.*, 1990).

It may be expected that mutations in p53 which are compatible with viability and normal early development must be 'weak' mutations which confer only a small growth advantage to the cell and which cannot act as dominant negative mutations. On the other hand, no such constraint is imposed on the somatically acquired mutations in tumours which might be expected to show a greater variation and include

**Figure 1** Sequence of the mutation.

Correspondence: J. Prosser.
Received and accepted 21 October 1991.

**Figure 2** Five generation pedigree showing incidence of cancer (black circles). Numbers below circles represent age at which cancer was diagnosed, age at death, current age, as appropriate. Arrow indicates the proband. Individuals tested for the p53 mutation are shown as wt/267 (carriers) and wt/wt (non-carriers).

'strong' mutations conferring considerable growth advantage. There is evidence (Milner & Medcalf, 1991) for the 'strength' of only one of the constitutionally mutated codons, 248, where a CGG->TGG mutation does not behave in a dominant-negative way when co-translated *in vitro* with wild-type p53 protein. This contrasts with the *in vitro* demonstration of the dominant-negative effect of proteins carrying various other sporadic mutations. Milner and Medcalf also looked at mutations at codon 273 (CGT->CCT and CGT->CTT) but did not investigate the particular recorded constitutional mutation at this site (CGT->CAG).

If we look at the overall frequency of total recorded mutations in those codons of p53 found constitutionally mutated (181, 242, 245, 248, 252, 258, 273, 282, 286, 307 and now 267), it is apparent that four of the sites are hypermutable (codons 245, 248, 273 and 282) where mutations account for approximately 25% of all those recorded in the p53 gene, codon 248 accounting for greater than 10% on its own (Caron de Fromentel & Soussi, 1991). Each of these sites contains a CpG dinucleotide (245 contains half a CpG dinucleotide) and 77% of the recorded mutations involve C->T changes at the CpG configuration. Although there is an under-representation of CpG dinucleotides in the vertebrate genome (Sved & Bird, 1990), it is known that 60–90% of them are methylated (Bird, 1986) and it is generally accepted that methylcytosine mutates at a high rate to thymine (Coulondre *et al.*, 1978). This type of spontaneous mutation, which occurs at 12 times the normal transition rate (Sved & Bird, 1990), is responsible for a high proportion of all p53 mutation found at these four hypermutable codons.

If we look only at the recorded constitutional mutations, half (10/19) occur at the four frequently mutated codons and nearly all of these (8/10) involve C->T mutations. Of the remaining nine (at codons 181, 242, 252, 258, 267, 286 and 307), two involve C->T changes at CpG dinucleotides. Three are at sites so far unique to constitutional mutations (181, 252) and the remaining six are at sites which are infrequently mutated (242, 258, 267, 286 and 307) and where the constitutional changes alone account for 53% of the recorded mutations.

In summary, more than half (10/19) of all constitutional p53 mutations appear to be spontaneous C->T changes at CpG dinucleotides and are frequently found at hypermutable sites (8/10). The codons of the remaining constitutional mutations are only infrequently mutated and have no consistent mutational pattern. (Five are C->T [G->A] in non-CpG dinucleotides, two are T->C [A->G], one is A->C [T->G], constituting seven transitions and one transversion, and one is loss of a single base.) The particular changes at the constitutional p53 mutations so far recorded are therefore consistent with the conclusion that endogenous spontaneous mutation could account for these events. The data are still too sparse to discuss 'hotspots' for constitutional mutations, but two codons (245 and 248) are responsible for eight of the 19 changes (42%). These are frequently mutated codons of the p53 gene in any case and are responsible for about 13% of all recorded mutations in the gene.

The authors would like to thank Mrs Rhona De Mey of the MRC Human Genetics Unit registry for construction and verification of the pedigree. Calculations of incidence of specific mutations in the p53 gene used data from Caron de Fromentel and Soussi (1991).

### References

BIRD, A.P. (1986). CpG-rich islands and the function of DNA methylation. *Nature*, **321**, 209.

CARON DE FROMENTEL, C.C. & SOUSSI, T. (1991). The TP53 tumour suppressor gene: a model for investigating human mutagenesis. In press in *Genes Chromes. Cancer.*

COTTON, R.G.H., RODRIGUES, N.R. & CAMPBELL, R.D. (1988). Reactivity of cytosine and thymine in single-base-pair mismatches with hydroxylamine and osmuium tetroxide and its application to the study of mutations. *Proc. Natl Acad. Sci. USA*, **85**, 4397.

COULONDRE, C., MILLER, J.H., FARABAUGH, P.J. & GILBERT, W. (1978). Molecular basis of base substitution hotspots in *Escherichia coli. Nature*, **274**, 775.

HOLLSTEIN, M., SIDRANSKY, D., VOGELSTEIN, B. & HARRIS, C.C. (1991). p53 mutations in human cancers. *Science*, **253**, 49.

LI, F.P. & FRAUMENI, J.F. (1969). Soft tissue sarcomas, breast cancer, and other neoplasms. *Ann. Intern. Med.*, **71**, 747.

MALKIN, D., LI, F.P., STRONG, L.C. & 8 others (1990). Germ line p53 mutations in a familial syndrome of breast cancer, sarcomas, and other neoplasms. *Science*, **250**, 1233.

METZGER, A.K., SHEFFIELD, V.C., DUYK, G., DANESHVAR, L., EDWARDS, M.S.B. & COGEN, P.H. (1991). Identification of a germ-line mutation in the p53 gene in a patient with an intracranial ependymoma. *Proc. Natl Acad. Sci. USA*, **88**, 7825.

MILNER, J. & MEDCALF, E.A. (1991). Cotranslation of activated mutant p53 with wild type drives the wild-type p53 protein into the mutant configuration. *Cell*, **65**, 765.

PROSSER, J., THOMPSON, A.L., CRANSTON, G. & EVANS, H.J. (1990). Evidence that p53 behaves as a tumour suppressor gene in sporadic breast tumours. *Oncogene*, **5**, 1573.

PROSSER, J., ELDER, P.A., CONDIE, A., MACFADYEN, L., STEEL, C.M. & EVANS, H.J. (1991). Mutations in p53 do not account for heritable breast cancer: a study in five affected families. *Br. J. Cancer*, **63**, 181.

SOUSSI, T., CARON DE FROMENTEL, C.C., STURZBECHER, H.W., ULLRICH, S., JENKINS, J. & MAY, P. (1989). Evolutionary conservation of the biochemical properties of p53: specific interaction of Xenopus laevis p53 with simian virus 40 large T antigen and mammalian heat shock proteins 70. *J. Virol.*, **63**, 3894.

SRIVASTAVA, S., ZOU, Z., PIROLLO, K., BLATTNER, W. & CHANG, E.H. (1990). Germ-line transmission of a mutated p53 gene in a cancer-prone family with Li-Fraumeni syndrome. *Nature*, **348**, 747.

SVED, J. & BIRD, A. (1990). The expected equilibrium of the CpG dinucleotide in vertebrate genomes under a mutational model. *Proc. Natl Acad. Sci. USA*, **87**, 4692.

VOGELSTEIN, B. (1990). A deadly inheritance. *Nature*, **348**, 681.